# F-Actin Dynamics in the Regulation of Endosomal Recycling and Immune Synapse Assembly

**DOI:** 10.3389/fcell.2021.670882

**Published:** 2021-06-24

**Authors:** Nagaja Capitani, Cosima T. Baldari

**Affiliations:** Department of Life Sciences, University of Siena, Siena, Italy

**Keywords:** vesicular trafficking, endosome, WASH complex, retromer, retriever, polarized recycling, actin dynamics

## Abstract

Membrane proteins endocytosed at the cell surface as vesicular cargoes are sorted at early endosomes for delivery to lysosomes for degradation or alternatively recycled to different cellular destinations. Cargo recycling is orchestrated by multimolecular complexes that include the retromer, retriever, and the WASH complex, which promote the polymerization of new actin filaments at early endosomes. These endosomal actin pools play a key role at different steps of the recycling process, from cargo segregation to specific endosomal subdomains to the generation and mobility of tubulo-vesicular transport carriers. Local F-actin pools also participate in the complex redistribution of endomembranes and organelles that leads to the acquisition of cell polarity. Here, we will present an overview of the contribution of endosomal F-actin to T-cell polarization during assembly of the immune synapse, a specialized membrane domain that T cells form at the contact with cognate antigen-presenting cells.

## Introduction

Surface expression of plasma membrane (PM)-associated receptors is dynamically regulated through constitutive or ligand-dependent endocytosis. Receptor internalization, which occurs in a clathrin-dependent or clathrin-independent manner (Doherty and McMahon, 2009), results in their targeting to the endocytic pathway. This pathway is orchestrated by a series of intracellular membrane-bound compartments that allow for the sorting of these molecules, referred to as cargoes, for one of two alternative fates: delivery to lysosomes or vacuoles for degradation by the endosomal sorting complex required for transport (ESCRT) and the multivesicular bodies (MVBs) compartment (Vietri et al., 2020) or targeting to the trans-Golgi network (TGN) or to the PM for reuse (Johannes and Wunder, 2011; Hsu et al., 2012). In this second route, the cargo is first recognized by a retrieval complex and routed away from the degradative pathway, then is pinched off from the endosome as a vesicle and coupled to cytoskeletal motor proteins for delivery to the target compartment (Burd and Cullen, 2014; Wang et al., 2018).

Sorting of endosomal cargo for recycling relies on a number of multiprotein complexes spatially and temporally regulated. The two main complexes responsible for endosomal retrieval are the retromer complex (Burd and Cullen, 2014) and the more recently identified retriever complex acting together with the CCC complex (Phillips-Krawczak et al., 2015; McNally et al., 2017). The Wiskott–Aldrich syndrome protein and SCAR homologue (WASH) complex plays essential roles in both retromer- and retriever-dependent pathways by promoting branched actin polymerization on endosomes (Gomez and Billadeau, 2009; Phillips-Krawczak et al., 2015; McNally et al., 2017). These complexes are not only important for the trafficking of molecules from the PM to the compartments of destination but also play a key role in polarized recycling of specific molecules to specialized areas of the cell as observed, for example, in T lymphocytes undergoing immune synapse (IS) formation. Indeed, upon T-cell receptor (TCR) engagement, endosomal trafficking is redirected toward the contact area of the T cell with the antigen-presenting cell (APC) by local F-actin pools that act in concert with microtubules and endosomal traffic regulators (Soares et al., 2013; Martín-Cófreces and Sánchez-Madrid, 2018; Onnis and Baldari, 2019). Here, we will review the role of the main molecular complexes involved in endosomal cargo recycling and the related actin dynamics, with a focus on polarized recycling to the T-cell IS.

## Sorting of Recycling Cargo at Early Endosomes by the Retromer, Retriever, and CCC Complexes

**FIGURE 1:**
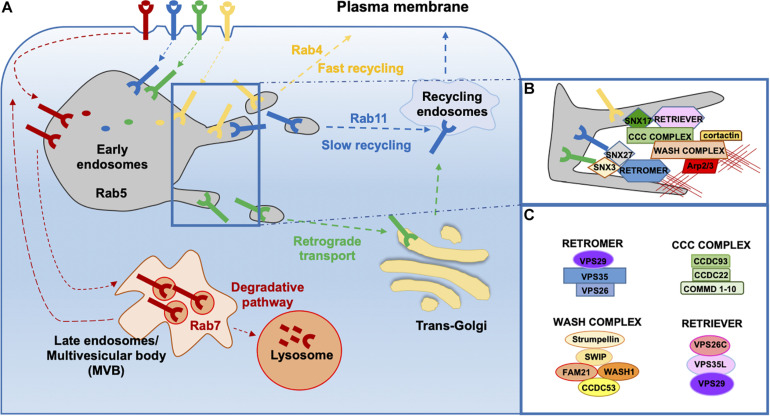
Overview of endosomal sorting and associated molecular machineries. **(A)** Following internalization, transmembrane proteins can be delivered to the degradative pathway through late endosomes and finally lysosomes or can be destined for recycling. Delivery of cargo back to the cell surface can occur directly through the fast recycling pathway or indirectly through the slow recycling pathway involving the pericentrosomal recycling compartment. Some cargo proteins can undergo retrograde transport from endosome to the Trans Golgi Network (TGN). **(B,C)** Multiprotein complexes involved in the retrieval and recycling of cargo proteins on **(B)** endosomes and the **(C)** components of each complex.

Endosomes are cellular hubs where internalized cargoes are sorted toward different trafficking pathways. Some cargoes are routed to the PM by recycling endosomes, a process known as endosome-to-plasma membrane recycling; others are transported to the TGN through an endosome-to-TGN retrieval or retrograde transport. Cargo recycling back to the cell surface can occur either via a fast recycling pathway controlled by the small GTPase Rab4 or via a slow recycling pathway in a Rab11-dependent manner (Galvez et al., 2012; Wandinger-Ness and Zerial, 2014; Figure 1A).

Endosomal sorting is accompanied by endosomal maturation. Early endosomes (EEs) are the main sorting station in the cell. EEs are characterized by specific markers such as Rab5 and early endosome antigen 1 (EEA1) and by the presence of large domains enriched in phosphatidylinositol(3,4,5)-triphosphate (PIP3) and sortin nexin (SNX) family members. A remarkable mosaicism in the EE membrane has emerged with the finding that cargoes, once they have reached the EEs, are targeted for degradation or recycling through the formation of specialized membrane subdomains that allow for cargo sorting and routing to the respective trafficking pathways through the local recruitment of specific molecular assemblies (Sönnichsen et al., 2000; Puthenveedu et al., 2010). The molecular machinery essential for cargo sorting in the recycling pathway is represented by three main complexes: retromer, retriever, and the CCC complex.

### Retromer

The retromer complex was first identified in *Saccharomyces cerevisiae* as a heteropentameric assembly consisting of a SNX heterodimer composed of vacuolar protein sorting (VPS), VPS5 and VPS17, and a heterotrimer composed of VPS26, VPS29, and VPS35, also known as “core” (Gallon and Cullen, 2015; Simonetti and Cullen, 2019; Figure 1C). SNXs are a large family of proteins containing a PX (phox homology) domain, which is responsible for binding to specific phosphoinositides (PIs) (Carlton and Cullen, 2005; Teasdale and Collins, 2012). In addition to the PX domain, SNXs may contain other domains and, on this basis, can be classified into five subfamilies: the SNX-PX subfamily, whose members are only endowed with a PX domain (e.g., SNX3) (Strochlic et al., 2007); the SNX-BAR subfamily, whose members comprise a BAR (Bin/Amphiphysin/Rvs) domain (e.g., the yeast VPS5-VPS17 and mammalian SNX1/2-SNX5/6) (Rojas et al., 2007; Wassmer et al., 2007); the SNX-FERM subfamily, whose members comprise PDZ (PSD95/Dlg/ZO) and FERM (protein 4.1/ezrin/radixin/moesin) domains (e.g., SNX27) (Temkin et al., 2011; Steinberg et al., 2013); the SNX–PXA–RGS–PXC (PX-associated domain A/regulator of G-protein signaling/PX-associated domain C) subfamily with a central PX domain flanked by several conserved domains (e.g., SNX13, SNX14, SNX19, and SNX25); and the SNX–MIT subfamily characterized by a microtubule interacting and transport domain (e.g., SNX15) (Teasdale and Collins, 2012). The core complex of retromer is conserved across all eukaryotes (Seaman, 2007), while the exact composition of the SNX dimer in mammals is less defined, with SNX1/SNX2 and SNX5/SNX6 as the mammalian orthologues of VPS5 and VPS17, respectively (Griffin et al., 2005; Wassmer et al., 2007). In mammals, the SNX dimer is responsible for the recruitment of the retromer to endosomes (Griffin et al., 2005), while the core complex is thought to participate in cargo binding and is therefore referred to as the “cargo recognition complex” (CRC) (Strochlic et al., 2007; Harterink et al., 2011; Temkin et al., 2011; Zhang et al., 2011; Steinberg et al., 2013).

Recent studies have revealed that SNXs play a central role in cargo recognition (Lucas et al., 2016). While some cargoes have been reported to directly bind to the CRC, recognition of other cargoes that recycle to the TGN or to the PM is mediated by SNX3 or SNX27 cargo adaptors, respectively (Strochlic et al., 2007; Harterink et al., 2011; Zhang et al., 2011). The retromer can directly interact with SNX3, resulting in the generation of a binding site for a canonical ØX(L/M) motif (where Ø is an aromatic amino acid) present in a variety of receptors, including the cation-independent mannose 6-phosphate receptor (CI-MPR) (Rojas et al., 2007; Seaman, 2007), the glycoprotein sortilin (Seaman, 2007; Canuel et al., 2008), the divalent metal transporter DMT1–II (Tabuchi et al., 2010), the G-protein-coupled receptor Wntless (Harterink et al., 2011; Zhang et al., 2011), and others. On the other hand, SNX27 recognizes, through its FERM and PDZ domains, the cytosolic domain of integral membrane proteins containing NPXY motifs or a carboxy-terminal class I PDZ-binding motif. Examples of this type of cargo include the β2 adrenergic receptor (β2AR), the glucose transporter GLUT1, the copper transporter ATP7A, and the glutamate receptors (Lauffer et al., 2010; Ghai et al., 2013; Steinberg et al., 2013; Gallon et al., 2014; McGough et al., 2014; Clairfeuille et al., 2016; Shinde and Maddika, 2017). Following retromer recruitment to endosomal membranes and cargo recognition via either SNX27 or SNX3, SNX-BAR proteins induce membrane deformation, generating endosomal tubules for cargo recycling to either the PM or the Golgi apparatus (Carlton and Cullen, 2005; Burd and Cullen, 2014; Figure 1B).

### Retriever

Not all cargoes transiting through the endosomal system require retromer for their trafficking (Steinberg et al., 2012; Kvainickas et al., 2017; Simonetti et al., 2017). Recently, McNally et al. (2017) identified and characterized a new protein complex, named retriever, required for sorting of a subgroup of transmembrane proteins. Retriever is a heterotrimer consisting of VPS26C (DSCR3), VPS35L (C16orf62), and VPS29, the latter shared with the retromer complex (Figure 1C). To fulfill its function in cargo recycling, the retriever, similar to retromer, needs to couple to an SNX protein, namely, SNX17 (Figure 1B). SNX17 interacts through its C-terminal tail with the VPS26C subunit of retriever, which is important for endosomal localization, while its FERM domain binds NPxY/NxxY motif-containing cargo proteins, such as the heterodimeric β1 integrins, the low-density lipoprotein receptor-related protein 1 (LRP1), the low-density lipoprotein receptor (LDLR), the epidermal growth factor receptor (EGFR), and others (Stockinger et al., 2002; Burden et al., 2004; Böttcher et al., 2012; Steinberg et al., 2012; Farfán et al., 2013; McNally et al., 2017). Interestingly, the interaction of retriever with SNX17 is not required for its association with endosomes. Similar to retromer, the retriever is not predicted to bind membranes; its endosomal recruitment depends on interactions with another complex, the CCC complex (McNally et al., 2017).

### The CCC Complex

The CCC complex consists of coiled-coil domain-containing proteins 22 (CCDC22) and 93 (CCDC93) and 10 members of the copper metabolism MURR1 domain-containing (COMMD) protein family (Maine and Burstein, 2007; Figure 1C). The CCC complex colocalizes with the retromer, retriever, and the WASH complex on endosomes (Phillips-Krawczak et al., 2015). CCC deficiency in human and mouse cells causes defective recycling of both SNX17/retriever-dependent (Bartuzi et al., 2016; McNally et al., 2017; Fedoseienko et al., 2018) and SNX27/retromer-dependent cargoes (Vonk et al., 2011; Phillips-Krawczak et al., 2015), indicating that CCC is required for both retromer- and retriever-dependent protein trafficking. Similar to retromer (Harbour et al., 2012), the CCC complex itself does not associate with endosomes but relies on its interaction with a component of the WASH complex, FAM21, for its correct localization (Phillips-Krawczak et al., 2015).

## The Wash Complex and Cortactin Coordinate F-actin Nucleation at Endosomes

The WASH complex is a pentameric complex composed of WASH1 (WASHC1), Strumpellin (WASHC5), the Strumpellin and WASH-interacting protein SWIP (also known as KIAA1033 or WASHC4), FAM21A/C (family with sequence similarity 21A and C, also known as WASHC2A/C), and coiled-coil domain containing protein 53 (CCDC53 or WASHC3) (Derivery et al., 2009; Gomez and Billadeau, 2009; Jia et al., 2010; Alekhina et al., 2017; Figure 1C). Among these, FAM21 is an important structural component of the WASH complex for its key role in interacting with other protein complexes through its long, unstructured C-terminal tail containing multiple functional binding sites consisting of 21 copies of the LFa motif, rich in leucine, phenylalanine, and several acidic residues (Derivery and Gautreau, 2010). FAM21 associates with multiple VPS35 retromer subunits (Harbour et al., 2012; Jia et al., 2012; Helfer et al., 2013), as well as with the CCDC93 subunit of the CCC complex (Phillips-Krawczak et al., 2015), thereby coupling both retromer and retriever to endosomes. FAM21 also contains two regions within its tail that are able to bind with intermediate affinity to PI3P and with strong affinity to PI(3,5)P2 (Singla et al., 2019). Moreover, FAM21 can associate with the CAPZα/β heterodimer, known as capping protein (CP). CP binds to the barbed end of the actin protofilament, thereby controlling filament growth by inhibiting monomer addition or loss from that end. FAM21 interacts directly with CAPZ and impairs its actin-capping activity (Hernandez-Valladares et al., 2010).

WASH1 is another important component of the WASH complex that serves as nucleation-promoting factor (NPF) by activating Arp2/3-dependent actin polymerization on endosomal membranes (Derivery et al., 2009; Gomez and Billadeau, 2009; Jia et al., 2010). Arp2/3 is a heptameric protein complex, so called because of its two main components, actin-related proteins (Arp) 2 and 3. It is the first actin nucleator identified in eukaryotic cells and is highly conserved among species (Pizarro-Cerdá et al., 2017). The ability of WASH to activate the Arp2/3 complex is finely tuned by ubiquitination (Hao et al., 2013). The E3 ubiquitin ligase TRIM27 and its enhancer MAGE-L2 are recruited by the retromer subunit VPS35 to WASH1, resulting in its K63-linked polyubiquitination, which leads to a conformational change that enhances actin nucleation (Hao et al., 2013). WASH ubiquitination is further regulated by the USP7 enzyme, which has a dual activity: to promote WASH ubiquitination by preventing TRIM27 auto-ubiquitination and degradation and, concomitantly, to limit WASH ubiquitination through its direct deubiquitination (Hao et al., 2015).

Another activator of the Arp2/3 complex that has been described to associate with endosomes is cortactin (Kaksonen et al., 2000; Lladó et al., 2008). Cortactin controls a wide range of processes including the maturation of late endosomes and lysosomes, the retrograde transport to the Golgi apparatus, and actin dynamics at endosomes. Cortactin is a class II NPF that promotes actin assembly both by inducing Arp2/3-dependent actin polymerization and by binding and stabilizing pre-existing branched F-actin nucleated by the WASH complex. Cortactin is in turn regulated by PI(3,5)P2, which directly interacts with its actin-binding domain, preventing F-actin binding and leading to the inhibition of cortactin-mediated branched F-actin nucleation and stabilization (Hong et al., 2015). Interestingly, the ability of PIs to regulate F-actin dynamics is not limited to PI(3,5)P2. For instance, PI(4,5)P2, the best characterized actin regulator, interacts with and modulates N-WASP and actin-binding proteins such as cofilin, CAPZ, filamin, vinculin, talin, and others (Yin and Janmey, 2003). On the other hand, PI(3,4,5)P3 regulates the activation of the WASP family member WAVE2 to control lamellipodial protrusion (Suetsugu et al., 2006), highlighting a pleiotropic role of PIs in actin cytoskeleton regulation (Saarikangas et al., 2010).

## Cytoskeletal Regulation of Endosomal Trafficking

**FIGURE 2:**
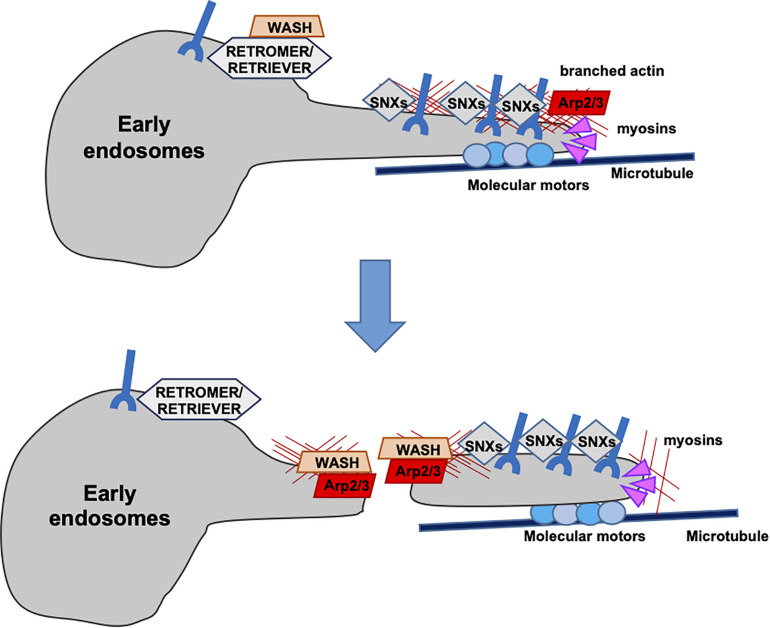
Recycling of receptors through actin-rich tubular domains. Sequence-dependent recycling of cargoes is tightly regulated by actin dynamics at the surface of endosomes. The retrieving machineries (retromer or retriever), together with the WASH complex, allow for the entry of the receptors into tubular profiles enriched in F-actin and actin-related proteins. Microtubule motors drive the stabilization and extension of the tubular profiles and branched F-actin contributes to vesicle fission.

Endosome sorting and maturation is accompanied by continuous membrane remodeling that requires the participation of both the actin and microtubule cytoskeletons. F-Actin is implicated in this process starting from the earliest steps, participating in defining endosomal subdomains to establish cargo destination. F-Actin associates with recycling microdomains and prevents the loss of recycling cargo to the degradative machinery (Simonetti and Cullen, 2019). In addition, F-actin polymerization at endosomes is essential for cargo sorting to recycling endosomes. Until recently, cargo recycling was believed to occur through sequence-independent “bulk” flow, as in the case of the transferrin receptor (Maxfield and McGraw, 2004). However, a large variety of cargoes have been demonstrated to recycle through a sequence-dependent pathway tightly regulated by actin dynamics (Puthenveedu et al., 2010; Temkin et al., 2011; Burd and Cullen, 2014). Cargoes to be recycled through this pathway are first recognized by a retrieval complex (retromer or retriever) and then packaged into tubulo-vesicular transport carriers enriched in F-actin and actin-related proteins (Puthenveedu et al., 2010). At this stage, microtubules join the game, with the microtubule-associated motor dynein and its partner dynactin, allowing for the extension of the nascent carriers. The WASH complex participates also in this step, exploiting its ability to interact with tubulin to stabilize the carriers on microtubule tracks. Subsequently, WASH-dependent F-actin nucleation at the apical portion of the tubular carriers provides the pushing force necessary for membrane fission. Following their detachment, F-actin regulates the short-distance mobility of tubular carriers mainly through actin motors such as myosin (Derivery et al., 2009; Harbour et al., 2010; Figure 2). This is exemplified by the β2AR receptor, which is recycled via tubular profiles enriched in F-actin and actin-related machineries (Bowman et al., 2016). A key role in this process is played by cortactin, which participates in the signaling cascade that regulates recycling (Vistein and Puthenveedu, 2014), the actin binding protein filamin A (FLNa) responsible for cargo entry into the tubular recycling domains (Pons et al., 2017), and other actin regulators including formins (Gong et al., 2018).

Following sorting at EEs, other recycling cargoes, such as the CI-MPR or sortilin, undergo retrograde trafficking to the TGN (Tu et al., 2020). In this process, cargoes are recognized by the SNX3–retromer complex, SNX-BARs, or clathrin and the adapter protein AP1, confined to specific endosomal subdomains and, following endosomal fission, transported by tubulo-vesicular transport carriers toward the TGN along microtubule tracks (Lu and Hong, 2014; Cheung and Pfeffer, 2016; Saimani and Kim, 2017). Once they arrive close to the TGN compartment, cargoes are captured by TGN-localized golgin proteins, such as golgin-97, golgin-245, GCC88, and GCC185 (Lowe, 2019). This process also involves TBC1D23, a protein that acts as a bridging protein by binding simultaneously to golgins and to the WASH complex subunit FAM21 on endosomal vesicles (Shin et al., 2017). Finally, carrier fusion with the Golgi membrane is mediated by four different vSNARE–tSNARE complexes (Lu and Hong, 2014).

## Interplay Between Rab GTPases and Molecular Complexes Implicated in Actin-Mediated Membrane Trafficking

Membrane trafficking is orchestrated by a variety of Rab GTPases that control different steps of the process, from cargo sorting to vesicle budding, motility, and fusion, through the recruitment of effector molecules, including the actin regulators mentioned in the previous paragraphs (Stenmark, 2009). Rab proteins are important regulators of retromer-mediated vesicular transport, not only in mammals but also in other organisms. For example, the core complex of retromer was found to interact with the GTP-bound form of Rab7 (Rab7-GTP) in yeast, plants, and mammalian cells (Rojas et al., 2008; Liu et al., 2012; Zelazny et al., 2013), leading to retromer recruitment to late endosomes (Priya et al., 2015). Rab32 regulates the retrograde trafficking of the CI-MPR to the TGN by directly interacting with SNX6 (Waschbüsch et al., 2019), while Rab21 is implicated in cargo sorting by establishing a complex with WASH and retromer to regulate endosomal F-actin (Del Olmo et al., 2019). Additionally, Rab9, together with retromer, WASH, and F-actin, has been recently reported to form an endosomal retrieval machinery that regulates selective recycling of the luminal protein Serpentine in the *Drosophila* trachea (Dong et al., 2013). Although little is known about the interplay between Rab GTPases and the retriever and CCC and WASH complexes, a recent proteomic study focused on SNARE and Rab proteins identified functional clusters, such as a correlation between Rab10 and the SNARE Syntaxin4 (STX4) or between Rab7/Rab21 and the WASH and CCC complexes (Clague and Urbé, 2020).

Rab GTPases also participate in tethering of the vesicles carrying recycling cargo to the target membrane through the recruitment of tethering factors (Stenmark, 2009). Rab32 and Rab38 were found to be implicated in the trafficking of the glucose transporter GLUT1 to the PM by regulating the effector molecule VARP, which in turn binds to the R-SNARE VAMP7 to facilitate membrane fusion between recycling endosomes carrying GLUT1 and the PM (Hesketh et al., 2014). SNX1 has been reported to interact with Rab6IP, a Rab6-interacting protein localized at the Golgi compartment and involved in the tethering of endosome-derived transport carriers to the TGN (Miserey-Lenkei et al., 2007). A similar function was observed for the *Drosophila* orthologue of TBC1D23, tbc1, a Rab GTPase-activating protein that couples endosome-derived vesicles to their target membrane at the TGN (Johnson and Andrew, 2019).

Although the specific identities and roles of Rab GTPases during receptor recycling are only beginning to be investigated, these findings underscore a tight functional interplay of these membrane trafficking regulators with the sorting, transport, and tethering machineries at all critical steps in receptor recycling. While the link between these Rabs and the cargo sorting complexes supports their implication in the local regulation of F-actin dynamics, the underlying mechanisms remain to be directly addressed.

## Actin Dynamics in Polarized Endosomal Trafficking: Focus on the Immune Synapse

The molecular complexes described above and the associated actin dynamics participate in cell polarization by regulating the recycling-dependent accumulation of receptors, adhesion molecules, and signaling mediators at specialized areas of the PM. This is exemplified by the formation of apical membrane specializations including primary cilia and apical microvilli of ciliated cells (Goldenring, 2015), the polarization of epithelial or neuronal cells (Vergés, 2016), or immune synapse (IS) formation in T lymphocytes and other immune cells.

The IS can be described as a highly polarized structure that forms at the T-cell interface with an APC carrying cognate MHC-bound antigen and allows the communication between the two cells to ensure efficient TCR signal transduction and T-cell activation (Dustin and Choudhuri, 2016). The typical “bull’s eye” structure of the mature IS features three concentric regions, referred to as supramolecular activation clusters (SMACs) that can be distinguished based on the specific partitioning of TCRs, costimulatory molecules, and integrins: the TCR-enriched central SMAC (cSMAC), the integrin-enriched peripheral SMAC (pSMAC), and the distal SMAC (dSMAC), where molecules with large ectodomains and negative regulators of TCR signaling are confined. IS assembly is coordinated by both the actin and the microtubule cytoskeletons, which drive the accumulation and partitioning of the synaptic components throughout the extended timeframe required for T-cell activation (Ritter et al., 2013; Martín-Cófreces and Sánchez-Madrid, 2018; Hammer et al., 2019).

The actin cytoskeleton plays a key role beginning from the first step of IS formation, which involves the assembly of TCR microclusters that move centripetally from the periphery to the center of the IS using F-actin as driving force (Ritter et al., 2013). In addition, actin promotes the activation of integrins to stabilize the T cell–APC contact and forms a ring-like seal at the inner side of the dSMAC (Hammer et al., 2019). However, the role of F-actin in IS assembly and function extends beyond the rearrangement and signaling events that occur at the PM. Early upon TCR activation, the centrosome translocates toward the IS in a process that is in part regulated by centrosomal F-actin dynamics (Ritter et al., 2013). Centrosome polarization is coordinated with F-actin clearance to generate a central F-actin-free area that facilitates the polarized release of effector molecules, such as cytokines produced by helper or cytotoxic T cells or the cytotoxic contents of the lytic granules of CTLs (Hivroz et al., 2012; Ritter et al., 2015; de la Roche et al., 2016; Kabanova et al., 2018; Herranz et al., 2019; Sanchez et al., 2019). Synaptic F-actin dynamics is also essential for the polarized release of vesicles carrying bioactive molecules, including synaptic ectosomes, which are assembled and released directly from the PM (Saliba et al., 2019), and exosomes, which are released upon the fusion of MVBs with the PM (Mittelbrunn et al., 2015; Bello-Gamboa et al., 2020).

Following polarization, the centrosome rapidly generates a network of microtubules both irradiating from the centrosome toward the periphery of the IS and converging from the periphery toward the center of IS to guide the polarized transport of vesicular components and organelles (Martín-Cófreces and Sánchez-Madrid, 2018). The identity of the vesicle-associated molecules that undergo polarized exocytosis to the IS depends on the type of T lymphocyte and APC, with helper T cells (CD4^+^ cells) secreting cytokines at the IS formed with cognate MHC-II-bearing cell targets to promote their maturation and function and cytotoxic T lymphocytes (CD8^+^ cells) releasing the toxic contents of their lytic granules at the IS formed with MHC-I-bearing cell targets for specific killing (Hivroz et al., 2012; de la Roche et al., 2016). Directional vesicular trafficking is, however, also the main mechanism by which T lymphocytes ensure the continuous availability of a functional pool of TCRs at the IS to sustain signaling during cell activation (Soares et al., 2013; Onnis et al., 2016; Finetti et al., 2017). This is achieved through the delivery to the synaptic membrane of TCRs associated with a pool of endosomes that undergo polarized recycling in a process regulated by both the tubulin and actin cytoskeletons (Martín-Cófreces and Sánchez-Madrid, 2018; Mastrogiovanni et al., 2020).

### TCR Endocytosis in Polarized Recycling to the IS

Receptor internalization is dependent on local actin polymerization to provide force for local membrane deformation and carrier budding (Hinze and Boucrot, 2018). The pathways that regulate both constitutive and ligand-dependent TCR internalization have been extensively investigated but are still debated (see Alcover et al., 2018 for an exhaustive coverage of TCR endocytosis). Two distinct endocytic routes of TCR endocytosis have been identified based on the requirement for the coat protein clathrin (Onnis and Baldari, 2019). In the clathrin-dependent endocytosis pathway, internalized TCRs are incorporated into a network of endosomal compartments defined by clathrin and the AP2 complex (Dietrich et al., 1994; von Essen et al., 2002; Crotzer et al., 2004). In the clathrin-independent endocytosis pathway, which appears as the main player in TCR recycling, internalized TCRs are incorporated into a dynamic endocytic network demarcated by the membrane-organizing proteins flotillins. Although flotillins are not required for TCR internalization, they are essential for the recycling of internalized TCRs to the IS and for full T-cell activation (Compeer et al., 2018). A third pathway involves the arrestin-dependent internalization of non-engaged, bystander TCRs for polarized recycling to the IS (Fernández-Arenas et al., 2014). Routing of TCR–CD3 complexes toward these alternative pathways of endocytosis and their subsequent targeting to recycling or late endosomes for subsequent degradation is dictated, at least in part, by the type of posttranslational modifications of the cytosolic domains of the CD3 complex components (Alcover et al., 2018).

### Cargo Sorting and Retrieval in Polarized Recycling to the IS

**FIGURE 3:**
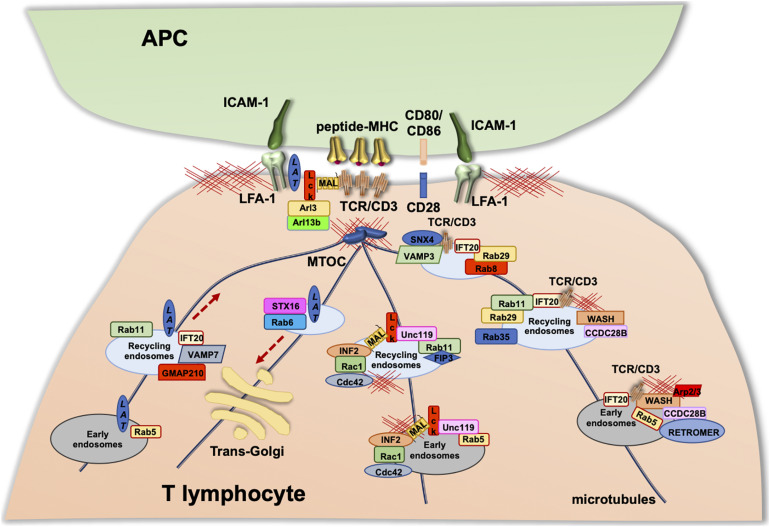
Vesicular trafficking in the regulation of IS formation. Upon TCR stimulation, the T-cell receptor, as well as associated signaling molecules (e.g., LAT and Lck), are delivered to the IS via endosomal vesicles. The polarized trafficking of different molecules occurs through distinct trafficking routes within the “classical” recycling pathway regulated by the Rab5 and Rab11. TCR-polarized trafficking to the IS involves additional Rab GTPases, some components of the intraflagellar transport (IFT) system, and the ciliogenesis protein CCDC28B that is essential for WAS-dependent actin polymerization on TCR^+^ endosomes. Lck associates with Rab11^+^ endosomal compartments, and its transport to the IS and sorting to the cSMAC are regulated by the uncoordinated 119 protein (Unc119), the membrane protein MAL, and Rab11-FIP3. LAT trafficking to the IS occurs through the classical Rab5 and Rab11 route, where anterograde transport is specifically regulated by GMAP210, IFT20, and VAMP7 and retrograde transport by Rab6 and Syntaxin-16.

Consistent with the role of F-actin in the process of endosome recycling described in the previous sections, proteins controlling actin polymerization and branching, such as the Arp2/3 component ARPC2 (Zhang et al., 2017) and WASH (Piotrowski et al., 2013), together with the retrieval complexes responsible for cargo recycling, regulate endosomal TCR trafficking and its polarization to IS. The interaction of retromer with WASH at EEs promotes F-actin nucleation, allowing for the generation of TCR carriers that undergo retrograde transport to the IS along microtubule tracks, to which they become coupled exploiting the tubulin-binding ability of WASH (Derivery et al., 2009; Gomez and Billadeau, 2009). We recently identified the ciliary protein coiled-coil domain containing 28B (CCDC28B) (Cardenas-Rodriguez et al., 2013), as a new component of the TCR retrieval machinery essential for polarized TCR recycling. We found that CCDC28B regulates actin polymerization at EEs carrying recycling TCRs by recruiting the FAM21–WASH complex to EE-associated retromer (Capitani et al., 2020; Figure 3). Consistent with the key role of WASH in regulating the recycling-dependent events occurring during IS formation, WASH deficiency in T lymphocytes results in a decrease in the surface levels not only of the TCR but also of the integrin LFA-1, the costimulatory receptor CD28, and the glucose transporter GLUT1 due to defective recycling (Piotrowski et al., 2013). Similarly, we observed defective TCR accumulation and signaling at the IS of CCDC28B-deficient cells downstream of early signaling and centrosome polarization caused by impaired TCR recycling (Capitani et al., 2020). Additionally, the Arp2/3 subunit ARPC1B was found to participate in IS formation in cytotoxic T lymphocytes by inducing receptor recycling to the PM via the retromer and WASH complexes. These include the TCR and the coreceptor CD8, as well as GLUT1 (Randzavola et al., 2019).

Recently, the retromer-associated SNX family member, SNX27, was found to be associated in resting T cells to early and recycling endosomes, largely through the interaction of its PX domain with PI3P. Upon T-cell engagement by APC, SNX27-enriched endosomes rapidly polarize toward the IS, accumulating at the cSMAC and pSMAC. The polarization of SNX27^+^ endosomes toward the IS is also regulated by PI binding, with the PX domain binding to PI3P-enriched membrane domains and the FERM domain to PI(4,5)P2- and/or PI(3,4,5)P3-enriched membrane domains (Rincón et al., 2011; Ghai et al., 2015). In support of the importance of PIs in SNX27 function, impaired PIP recognition by the SNX27 FERM domain affected its localization at the endosomal recycling compartment and impaired its correct distribution during initial steps of IS formation (Tello-Lafoz et al., 2014). In addition, proteomic analysis of the SNX27 interactome in activated T cells confirmed that SNX27-mediated trafficking involves the retromer and WASH complexes and also revealed additional cargoes that associate with SNX27 in polarized recycling to IS (Tello-Lafoz et al., 2017). Among these are the lipid second messenger diacylglycerol (DAG) (Rincón et al., 2007); the protein zonula occludens-2 (ZO-2), a tight junction scaffold protein recently identified in T lymphocytes; the centromere protein J (CENPJ), which acts as a microtubule plus-end tracking protein; and the Rho guanine nucleotide exchange factor 7 (ARHGEF7) (González-Mancha and Mérida, 2020). These results indicate that other receptors or membrane-associated signaling components that participate in IS assembly may exploit the retromer-regulated pathway for EEs sorting and redirection to the synaptic membrane.

While a role for retromer in IS formation is well established, less is known about the contribution of retriever and the CCC complex to this process, although the identification of retriever-associated SNX17 bound to TCR complexes at the IS suggests that multiple retrieval complexes coexist during polarized recycling of endosomal synaptic components. Interestingly, SNX17 silencing affects TCR and LFA-1 expression at the T-cell surface, suggesting that SNX17 is required to maintain functional surface pools of activating receptors and integrins to allow for IS formation and T-cell activation (Osborne et al., 2015).

### Multiple Recycling Pathways Regulate IS Assembly

In addition to the TCR, two membrane-bound molecules essential for TCR signaling also undergo polarized recycling to the IS: the initiating lymphocyte-specific protein tyrosine kinase Lck (Ehrlich et al., 2002) and the transmembrane adaptor linker for activation of T cells (LAT) (Bonello et al., 2004). Similar to the TCR, these molecules form two different pools within the cell, one associated with the plasma membrane and the other with the endosomal and Golgi compartments, which are sequentially delivered to the IS to sustain signaling (Figure 3). The polarized trafficking of these molecules occurs through distinct routes within the “classical” recycling pathway regulated by the Rab5 and Rab11. Polarized TCR recycling to the IS involves additional Rab GTPases, which include Rab8, Rab29, and Rab35 (Finetti et al., 2015; Onnis et al., 2015; Patino-Lopez et al., 2018) and the intraflagellar transport (IFT) system components IFT20, IFT54, IFT57, and IFT88 (Finetti et al., 2009, 2014). Similar to the TCR, Lck associates with Rab11^+^ endosomal compartments, and its transport to the IS and sorting to the cSMAC is regulated by a variety of molecules. Among these are the uncoordinated 119 protein (Unc119), which extracts PM-bound Lck by sequestering its hydrophobic myristoyl group and releases the kinase at the synaptic membrane under the control of the ARL3/ARL13B complex (Stephen et al., 2018); the membrane protein MAL that, together with the formin INF2, generates specific carriers for Lck targeting to the IS in a Cdc42-Rac1-dependent manner (Andrés-Delgado et al., 2010; Antón et al., 2011); and the Rab11 effector FIP3 (Rab11 family interacting protein-3), which plays a key role in the regulation of the subcellular localization and function of Lck (Bouchet et al., 2017). LAT trafficking to the IS occurs through the classical Rab5 and Rab11 route, where anterograde transport is specifically regulated by the golgin GMAP210, the intraflagellar transport protein IFT20 and the SNARE VAMP7 and retrograde transport by Rab6 and SNARE Syntaxin-16 (Larghi et al., 2013; Vivar et al., 2016; Carpier et al., 2018; Zucchetti et al., 2019; Saez et al., 2021).

Although the role of endosomal actin in IS assembly and T-cell activation has been well established, the underlying mechanisms remain to be fully understood. As detailed in *Cargo sorting and retrieval in polarized recycling to the IS*, actin polymerization at TCRs undergoing sorting at early endosomes is mediated by both the retromer and retrieval complexes through the WASH-dependent recruitment of Arp2/3. CCDC28B plays a key role in this process by coupling the FAM21 component of the WASH complex to the retromer at endosomes carrying recycling TCRs (Capitani et al., 2020). Interestingly, Rab11^+^ endosomes indirectly regulate actin dynamics at the synaptic membrane by allowing for the polarized transport of the Rac GTPase Rac1, which associates with Rab11 through its effector FIP3 (Bouchet et al., 2016, 2018). MVBs that are delivered to the synaptic membrane also contribute to local actin polymerization through the clathrin-dependent recruitment of proteins that are implicated in this process, such as dynamin-2, Arp2/3, and CD2AP (Calabia-Linares et al., 2011). In this emerging scenario, endosomal and plasma membrane actin dynamics establish a tight interplay to sustain IS architecture and signaling during T-cell activation.

## Conclusion

The striking architecture of the IS was described over 20 years ago. Not surprisingly, rearrangements of TCRs, adhesion molecules, costimulatory receptors, and membrane-associated signaling mediators occurring at the region of the T-cell plasma membrane at the contact with cognate APC have been extensively investigated. Only more recently has vesicular trafficking entered the picture with the finding that intracellular pools of the TCR and other components of the IS play a key role in this process beyond the known function of polarized delivery of effectors in differentiated T cells. From the initial finding that intracellular TCRs are delivered to the synaptic membrane through polarized recycling, it has become clear that the TCR is by no means unique in this respect. A wide array of other membrane-associated molecules, including receptors such as CD28 and signaling mediators such as Lck and LAT, have been demonstrated to undergo polarized recycling (Onnis and Baldari, 2019). Strikingly, with the identification of new regulators of the traffic of these molecules, achieved with essential input from the fields of vesicular trafficking and ciliogenesis (Cassioli and Baldari, 2019), it is now clear that a diversity of recycling pathways characterized by unique combinations of Rab GTPases and respective GEFs, v- and t-SNAREs, and tethering proteins coexist within the classical recycling pathways defined by Rab11 (Onnis et al., 2016).

While a role for microtubules and microtubule motors for the movement of these endosomes was expected, the identification of endosomal F-actin as a key player in the sorting of recycling molecules and their coupling to microtubules has brought a new layer of complexity to the process of polarized recycling to the IS. The identification of the retromer, retriever, and the CCC complex as a different means to achieve the polymerization of new actin filaments at endosome subdomains enriched in specific receptors that are destined for recycling has highlighted a diversity also at this step of the pathway (Wang et al., 2018; Simonetti and Cullen, 2019). Further work will be required to unravel the mechanisms and molecular machineries responsible for the specificity in the selection of both the receptors to be sorted for recycling and the respective actin-nucleating complex on which their transit from early to recycling endosomes depends. Additionally, how membrane subdomains are generated at early endosomes to serve as hubs for the accumulation of individual receptors and associated regulators remains elusive, as do the fine details of local force generation for the abscission of vesicles that will mature to recycling endosomes. We expect that a multidisciplinary approach to these questions capitalizing on converging new knowledge and new technologies gleaned from immunology, cell biology, and biophysics will be crucial to unravel the increasing complexity of the process of IS assembly.

## Author Contributions

NC and CB wrote the manuscript. NC prepared the artwork. Both the authors contributed to the article and approved the submitted version.

## Conflict of Interest

The authors declare that the research was conducted in the absence of any commercial or financial relationships that could be construed as a potential conflict of interest.
